# A Novel Split-Course High-Dose Palliative Radiotherapy Regimen for Locally Advanced Sinonasal Cancer: A Case Report

**DOI:** 10.1155/2024/9340657

**Published:** 2024-10-03

**Authors:** Saif Aljabab, Amna Mohaimeed, Firas AlMomen, Abdullah AlSwailem, Yasir Alayed

**Affiliations:** ^1^ Radiation Oncology Unit College of Medicine King Saud University, Riyadh, Saudi Arabia; ^2^ Oncology Center King Saud University Medical City, Riyadh, Saudi Arabia

**Keywords:** head and neck cancer, palliative radiotherapy, sinonasal cancer

## Abstract

Sinonasal malignancies (SNMs) are rare heterogeneous malignancies that frequently present with locally advanced disease. The prognosis is poor when the disease is considered extensive and unresectable. In such cases, a high-dose palliative radiotherapy regimen is often required, but the ideal dose and fractionation have not been established. We detail a 33-year-old male who initially presented with a progressively growing mass over the right cheek. A biopsy of the lesion revealed squamous cell carcinoma (SCC). Imaging revealed a very advanced and unresectable disease with the involvement of several head and neck subsites. He progressed further after receiving induction chemotherapy from an outside institution. The patient requested prompt tumor and symptom control to travel back to his home country. We offered him high-dose split-course palliative radiotherapy in the form of a quad Shot of 14.80 Gy in four fractions twice daily, followed by 30 Gy in five fractions every other day with a 2-week interval. Treatment resulted in excellent clinical response with symptomatic relief in a short time, and the patient could travel back home safely.

**Summary**

This case report describes a novel high-dose palliative radiotherapy regimen in a patient diagnosed with locally advanced unresectable sinonasal squamous cell carcinoma (SCC). The patient was offered a split-course high-dose palliative RT within a 2-week interval, with excellent clinical response and symptomatic relief.

## 1. Introduction

Sinonasal malignancies (SNMs) are rare, accounting for less than 1% of cancer diagnoses and less than 3% of all head and neck cancer (HNC) diagnoses [1]. They are more predominant in males, with most cases occurring in the fifth to seventh decade of life. The most common primary site is the nasal cavity (40%–50%), followed by the maxillary sinus (30%–40%). The most predominant histological subtypes are squamous cell carcinoma (SCC) and adenocarcinoma, accounting for 51.6% and 12.6%, respectively [2]. SNMs are associated with multiple occupational risk factors, including wood dust, leather, heavy metals, glues, and formaldehyde. Other nonoccupational risk factors include smoking, alcohol consumption, nasal polyposis, and chronic sinusitis [2]. Evidence suggests that human papillomavirus (HPV) has been detected in 30% of all SNM and is considered a favorable prognostic factor [3].

Patients with SNM present with various unspecific symptoms, including ear fullness, nasal congestion, headache, facial swelling, and pain [4, 5]. Due to the aggressive nature of these tumors, most patients present in advanced stages. The prognosis is poor, with a 5-year overall survival (OS) of 45.7% [6]. Standard workup includes medical history, physical examination, computed tomography (CT), magnetic resonance imaging (MRI) with contrast, and fluorodeoxyglucose-positron emission tomography (FDG-PET-CT) scans [7]. The primary treatment for SNM is surgical resection. Advanced resectable disease is managed with a combination of surgery and radiotherapy, typically given in the adjuvant setting [8]. For nonresectable tumors, definitive radiotherapy with or without concurrent chemotherapy can be offered [9]. Multimodality treatment is often employed and is associated with improved survival in retrospective series [10]. However, the SINTART trial was the first prospective, nonrandomized trial on combined-modality treatment, which reported no significant improvement in survival rates with multimodality treatment [11].

Palliative treatment for advanced head and neck disease can offer remarkable symptomatic relief. Different palliative radiation regimens are described in the literature, including the “quad Shot” regimen, which is a cyclical hypofractionated regimen with doses ranging between 3.5 and 3.7 Gy per fraction given twice a day on two consecutive days, with a minimum of 6-h interval between the two fractions. This regimen can be delivered every 3–4 weeks up to a total of three cycles [12]. There is emerging evidence that hypofractionated regimens result in excellent objective response, symptomatic relief, and improved quality of life [12]. In this case report, we describe a novel high-dose split-course palliative regimen for a patient with advanced unresectable maxillary cancer.

## 2. Case Report

We detail a 33-year-old male who presented in January 2022 to a small private hospital with a fungating, erythematous, progressive, and ulcerative mass measuring approximately 5 × 4 cm over the right cheek area. He had a good performance status (ECOG 1) with an unremarkable past medical and surgical history. The patient was a nonsmoker who worked at a local supermarket. He was from a foreign country with limited local support and no nearby family or friends. Initial MRI and FDG-PET-CT findings revealed a right maxillary sinus aggressive lesion with inferior orbital wall extension measuring 6.5 cm × 5 cm as well as a moderate FDG activity within the ipsilateral cervical lymph node measuring 1.6 cm in Level II, which was most likely metastatic. An outside biopsy of the mass revealed SCC. The patient was evaluated by an external head and neck surgeon and medical oncologist who decided that it was unresectable and requested surgical re-evaluation after induction chemotherapy with docetaxel, cisplatin, and fluorouracil (TPF) for three cycles.

**Figure 1:**
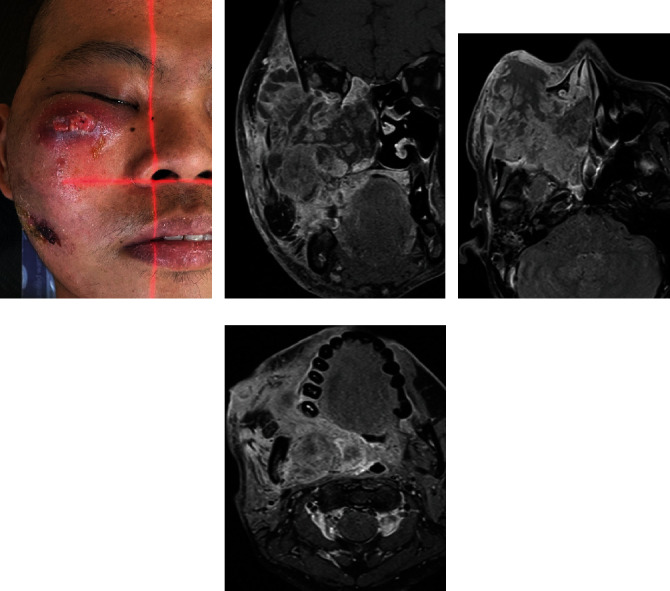
Patient's initial presentation at our facility before first treatment. (a) The patient's clinical picture was taken at the time of CT simulation. (b) Coronal view of the disease on MRI T1 postcontrast imaging. (c) Axial view, level of the orbit. (d) Axial view, level of the mandible.

Several months later, in May 2022, the patient was referred to our institution where he was evaluated by a multidisciplinary head and neck team for a second opinion. Upon presentation, his local examination revealed an enlarging right fungating cheek mass, indicating disease progression. The tumor displaced the right eye with conjunctivitis but no visual loss. Ear examination showed right tympanic membrane petechia, with decreased hearing to soft sounds. The oral cavity exam was limited due to trismus. A neurological exam revealed no cranial nerve palsies. Local restaging with an MRI revealed interval disease progression manifested by the increased size of the right-sided sinonasal cavity tumor measuring 9 × 8 × 7 cm, with more extension to adjacent structures including the right-sided maxillary sinus, ethmoid air cells, sphenoid sinus, frontal sinus, pterygopalatine fossa, sphenopalatine foramen, right prevertebral region, skull base foramina, cavernous sinus, parapharyngeal space, supra zygomatic part of the masticator space, and temporalis muscle. In addition, there was an extension to the lateral and inferior extracoronal compartment of the right orbital cavity, oral cavity, right oropharynx, right nasopharynx, and superior supraglottic larynx. Right-sided cervical nodes were unchanged, and there were no intracranial, brainstem, or brain parenchyma extensions. Final staging was T4bN1M0, Stage IVB (AJCC 8th edition). Images of the patient and MRI are outlined in Figure 1. A central pathology review of the tumor biopsy confirmed the diagnosis of moderately differentiated SCC. The case was discussed in a multidisciplinary meeting, the disease was rendered incurable, and the board advised a percutaneous endoscopic gastrostomy (PEG) tube insertion and palliative radiotherapy.

**Figure 2:**
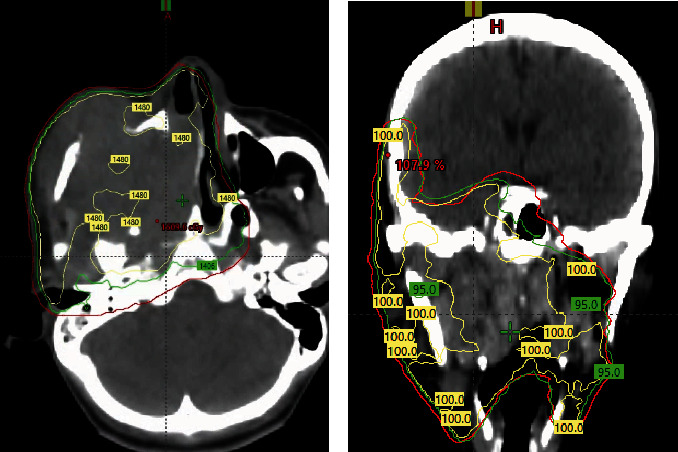
Initial radiotherapy regimen of 14.8 Gy BID over 2 days. (a) Axial view of final plan. (b) Coronal view of final radiotherapy plan; 100% represents the prescription dose of 14.8 Gy.

**Figure 3:**
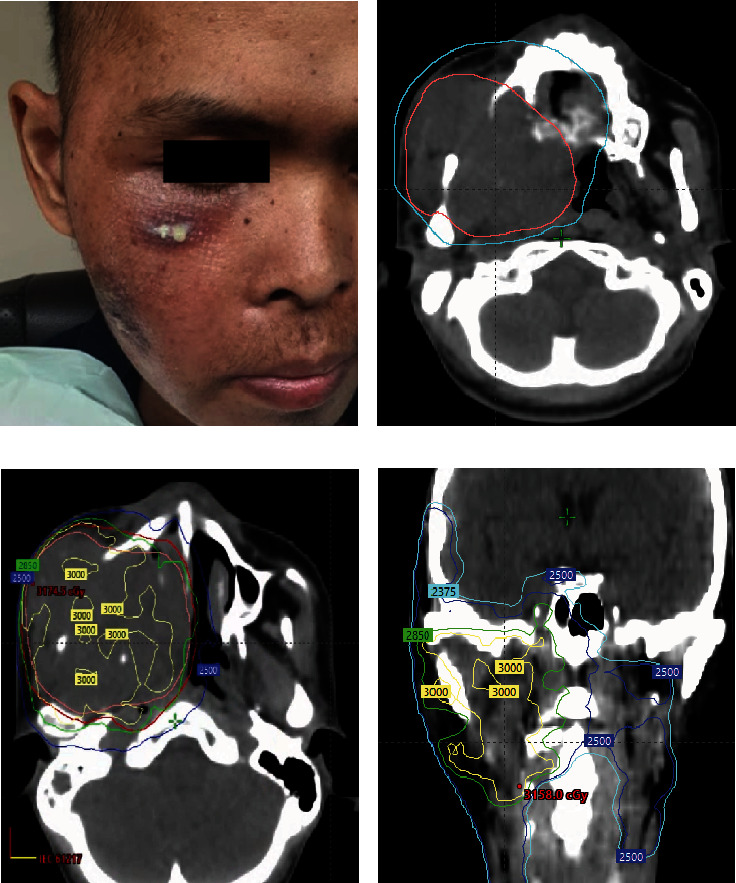
Two weeks posttreatment evaluation and second radiotherapy of 30 Gy in five fractions delivered every other day. (a) Clinical picture of the patient before the second treatment reveals excellent clinical response. (b) Axial view of CT scans showing a reduction in the size of the initial tumor (cyan) and current tumor (pink). (c) Axial view of the second radiotherapy plan. (d) Coronal view of the second radiotherapy plan.

**Figure 4:**
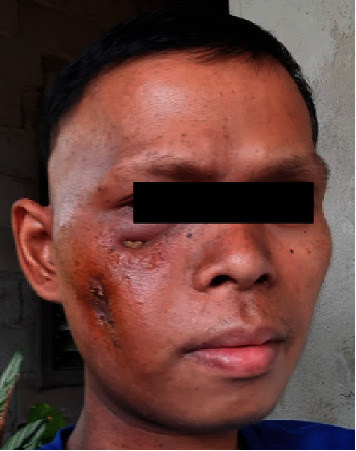
Photo of the patient 2 months posttreatment from the Philippines revealing excellent clinical response.

At this stage, the patient wished to complete treatment as soon as possible, preferably within a month, in order to travel safely to his home country. Our initial decision was to proceed with the standard quad Shot regimen. However, the standard timeline of this regimen was not appealing to the patient. The challenge presented was to do a high-dose palliative regimen in a short period of time for the patient to be healthy enough to travel back home within a month. We initiated treatment with a quad Shot radiotherapy regimen with a total dose of 14.8 Gy in four fractions, given twice a day (Figure 2). Instead of repeating the quad Shot in 3–4 weeks, we followed by a quick re-evaluation in 2 weeks and a hypofractionated regimen of 30 Gy in five fractions every other day (Figure 3). Planning was done on a standard linear accelerator (TrueBeam, Varian) using a 6-MV volumetric modulated arc therapy (VMAT) technique, the gross tumor volume (GTV) was defined as all gross visible disease on fused MRI imaging, and an additional 3 mm margin was added to create the planning target volume (PTV). We did not pursue any elective or clinical target volume (CTV) in either plan. Both regimens were administered in June 2022 for the patient to travel within the following month. Postradiotherapy, the tumor responded well to treatment, and his pain, dysphagia, and trismus improved dramatically. The patient managed to travel back home and contact us a month later where he continued to do well (Figure 4). Unfortunately, further follow-up with the patient was limited as he lived in a remote area in the Philippines, and we received a second-hand report that the patient passed away nearly 6 months later, and the cause of death was not clearly reported.

## 3. Discussion

Maxillary sinus tumors are the second most common primary site of SNM, preceded by the nasal cavity [1]. The mainstay of treatment is maximal surgical resection, with radiotherapy mostly employed in the adjuvant setting in cases with adverse pathologic features such as T3/T4 disease, positive margins, and extensive nodal disease [8]. The presenting symptoms of SNM are usually vague, leading to a delay in diagnosis for the majority of patients [1]. Management requires a multidisciplinary approach including experts in HNCs, which is often scarcely available in underserved regions.

In 1980, palliative radiotherapy in the form of quad Shot protocol was first described in RTOG 85-02 for the treatment of pelvic malignancies. This protocol allowed for lower acute and late toxicities and better tumor response as compared to the historical RTOG 7905 protocol, which was a cyclical hypofractionated regimen of 10 Gy in a single fraction to the whole pelvis every 4 weeks [13, 14]. Subsequently, the quad Shot protocol was incorporated into the treatment of advanced HNCs alongside other sites. Published evidence on different palliative radiotherapy regimens for head and neck malignancies is mostly retrospective in nature, making it challenging to draw any strong conclusions about the optimal treatment approach. The implementation of shorter treatment schedules that deliver higher doses improves compliance and has the potential to improve outcomes, particularly by addressing the challenge of accelerated repopulation [15]. Hypofractionated radiotherapy offers several advantages in the palliative setting for HNCs. This includes rapid tumor response, minimal acute and late toxicity, decreased hospital visits, increased compliance, excellent objective response, and potentially improved OS [16]. A single institution review for incurable HNCs using a quad regimen with VMAT technique concluded an overall tumor response and symptomatic relief in 94% of patients [17]. Another prospective study from India reviewed the quad regimen revealing a survival benefit after completion of three cycles (MS 7 months) when compared to two cycles (MS 3 months). Notably, some patients who received three cycles survived more than 1.5 years [18]. Ghoshal et al. included patients with Stage III and IV HNCs treated with 30 Gy in 10 fractions. They reported > 50% symptom improvement in 90% of patients. Pain relief persisted for at least 3 months in 64% of the patients, and no Grade 3 toxicities were observed [19]. The various palliative radiotherapy regimens for locally advanced HNCs are summarized in Table 1.

The objective of this case report was to describe a novel high-dose split-course palliative radiotherapy regimen for very advanced SNM patients requiring treatment within a short time interval. In this case, the sinonasal mass was very advanced and incurable due to the aggressive nature of the disease, delayed diagnosis, and resistance to initial treatment. A total dose of 14.80 Gy BID over 2 days followed by 30 Gy in five fractions after a 2-week break resulted in significant objective tumor response with good tolerance to treatment and symptom improvement such as decreased pain. The rationale behind the split course was to achieve an initial tumor volume reduction to allow for the delivery of a more hypofractionated regimen that can meet dose constraints. The patient managed to return home safely and continued to do well before succumbing to his disease several months later. These findings are restricted to a single patient with limited follow-up; however, the following case report suggests a good response to a novel short-interval split-course radiotherapy palliative regimen for patients with very advanced incurable disease. We plan to further explore this approach in a small clinical study.

## 4. Conclusion

A short-interval split-course high-dose palliative radiotherapy regimen of 14.8 Gy BID over 2 days followed by 30 Gy in five fractions provided an excellent tumor response and symptom relief in a case with very advanced and unresectable sinonasal tumor.

## Figures and Tables

**Table 1:** Summary of studies including high-dose palliative radiation for head and neck cancer.

**Author**	**Year**	**Type**	**n**	**Regimen**	**Response rate**	**MS (mo.)**	**Grade 3+ toxicity (%)**
Minatel et al. [20]	1998	Prospective	62	25 Gy/10 fx, OD (given twice with a 2-week break) with concurrent bleomycin	28% CR 41% PR 81% symptom palliation	7	43% G3 mucositis
Corry et al. [21]	2005	Prospective	30	Three courses of 14 Gy/4 fx, BID, Q4 weeks	6% CR 47% PR 44% QOL improvement	5.7	0% G3 mucositis
Porceddu et al. [22]	2007	Prospective	35	30 Gy/5 fx, OD, twice weekly	56% CR 9% PR 67% pain improvement	6	26% G3 mucositis 11% G3 dysphagia
Chen et al. [23]	2008	Retrospective	23 13 12 7 5	14.8/4 fx, BID up to 3 courses 70 Gy/35 fx, OD 30 Gy/10 fx, OD 37.5 Gy/15 fx, OD 20 Gy/5 fx, OD	83% 77% 67% 86% 60%	4 6 8 5 3	9% G3 toxicity 38% G3 toxicity 42% G3 toxicity 29% G3 toxicity 20% G3 toxicity
Nguyen et al. [24]	2015	Retrospective	110	24 Gy/3 fx, 1 fraction/week	31% CR 50% PR 82% symptom palliation	6.2	1% G3 mucositis
Straube et al. [25]	2016	Retrospective	27	40 Gy/20 fx, OD SIB to 54 Gy/20 fx	65%	6	41%
Fortin et al. [26]	2016	Prospective	32	25 Gy/5 fx, OD	—	6.5	13% any G3 toxicity
Murthy et al. [27]	2016	Prospective	126	32 Gy/8 fx, OD, twice weekly	3.2% CR 41% PR 76% symptom palliation	5.5	1.2% G3 mucositis

Abbreviations: BID: twice daily; CR: complete response; MS: median survival; *n*: number of patients; OD: once daily; PFS: progression-free survival; PR: partial response; QoL: quality of life; RR: relative risk; SD: stable disease; SIB: simultaneous integrated boost.

## Data Availability

Access to data is restricted due to third-party rights and patient privacy.
